# Analysis of *PTPN22* −1123 G>C, +788 G>A and +1858 C>T Polymorphisms in Patients with Primary Sjögren’s Syndrome

**DOI:** 10.3390/diagnostics13050899

**Published:** 2023-02-27

**Authors:** Paula Annahi Menchaca-Tapia, Miguel Marín-Rosales, Diana Celeste Salazar-Camarena, Alvaro Cruz, Edith Oregon-Romero, Raziel Tapia-Llanos, José Francisco Muñoz-Valle, Claudia Azucena Palafox-Sánchez

**Affiliations:** 1 Doctorado en Ciencias Biomédicas, Centro Universitario de Ciencias de la Salud, Universidad de Guadalajara, Guadalajara 44340, Mexico; 2 Instituto de Investigación en Ciencias Biomédicas, Centro Universitario de Ciencias de la Salud, Universidad de Guadalajara, Guadalajara 44340, Mexico; 3 Servicio de Reumatología, Hospital General de Occidente, Secretaria de Salud Jalisco, Guadalajara 45170, Mexico; 4 Grupo de Inmunología Molecular, Centro Universitario de Ciencias de la Salud, Universidad de Guadalajara, Guadalajara 44340, Mexico

**Keywords:** *PTPN22* polymorphisms, primary Sjögren’s Syndrome, *PTPN22* expression

## Abstract

Background: Primary Sjögren’s syndrome (pSS) is an autoimmune exocrinopathy characterized by lymphocytic infiltration, glandular dysfunction and systemic manifestations. Lyp protein is a negative regulator of the T cell receptor encoded by the *tyrosine phosphatase nonreceptor-type 22* (*PTPN22*) gene. Multiple single-nucleotide polymorphisms (SNPs) in the *PTPN22* gene have been associated with susceptibility to autoimmune diseases. This study aimed to investigate the association of *PTPN22* SNPs rs2488457 (−1123 G>C), rs33996649 (+788 G>A), rs2476601 (+1858 C>T) with pSS susceptibility in Mexican mestizo subjects. Methods: One hundred fifty pSS patients and 180 healthy controls (HCs) were included. Genotypes of *PTPN22* SNPs were identified by PCR-RFLP. *PTPN22* expression was evaluated through RT–PCR analysis. Serum anti-SSA/Ro and anti-SSB/La levels were measured using an ELISA kit. Results: Allele and genotype frequencies for all SNPs studied were similar in both groups (*p* > 0.05). pSS patients showed 17-fold higher expression of *PTNP22* than HCs, and mRNA levels correlated with SSDAI score (*r*^2^ = 0.499, *p* = 0.008) and levels of anti-SSA/Ro and anti-SSB/La autoantibodies (*r*^2^ = 0.200, *p* = 0.03 and *r*^2^ = 0.175, *p* = 0.04, respectively). Positive anti-SSA/Ro pSS patients expressed higher *PTPN22* mRNA levels (*p* = 0.008), with high focus scores by histopathology (*p* = 0.02). Moreover, *PTPN22* expression had high diagnostic accuracy in pSS patients, with an AUC = 0.985. Conclusions: Our findings demonstrate that the *PTPN22* SNPs rs2488457 (−1123 G>C), rs33996649 (+788 G>A) and rs2476601 (+1858 C>T) are not associated with the disease susceptibility in the western Mexican population. Additionally, *PTPN22* expression may be helpful as a diagnostic biomarker in pSS.

## 1. Introduction

Primary Sjögren’s syndrome (pSS) is an autoimmune disease characterized by lymphocyte infiltration to lachrymal and salivary glands and impaired secretory activity, leading to the most important manifestations of the disease, keratoconjunctivitis *sicca* and xerostomia [1]. The etiology of this disease is incompletely understood; however, a key element in the pathogenesis is T and B lymphocyte hyperactivity, leading to autoantibody production mainly against ribonucleoproteins (SSA/Ro and SSB/La) and consequent presence of hypergammaglobulinemia [2,3]. It has been suggested that pSS is a complex and multifactorial disease, with genetic, environmental and hormonal factors involved in the disease pathogenesis. The *protein tyrosine phosphatase nonreceptor type 22* (*PTPN22*) gene encodes the cytoplasmic protein lymphoid tyrosine phosphatase protein (Lyp), a potent downregulator of T cells, by inhibiting signaling through dephosphorylation of several substrates [4]. *PTPN22* is involved in calibrating the T cell activation threshold and terminating TCR signaling [5].

Diverse case-control studies have examined the potential contribution of *PTPN22* SNPs and their haplotypes to susceptibility to different autoimmune diseases (AIDs); however, results are inconsistent, in part because of ethnic and racial differences [6,7,8,9]. For example, rs2488457 (−1123 C) has been associated with type 1 diabetes mellitus in the Korean population [10]. In the Chinese population, rs2488457 is associated with rheumatoid arthritis (RA) [11], latent autoimmune diabetes in adults [12] and ulcerative colitis (UC) [13], whereas it is reported to be associated with less risk of systemic lupus erythematosus (SLE) in the Mexican population [14]. In addition, Muñoz-Valle et al. found an association between rs2488457 and lower levels of anti-citrullinated antibodies in RA patients [15].

The SNP rs33996649 (+788 G>A) is located in region encoding the catalytic domain of Lyp and represents a change in arginine (R) to glutamine (Q) (R263Q). This amino acid alteration leads to loss of function through reduced phosphatase activity [7]. rs33996649GA has also been related to protection against autoimmune diseases in European and American populations [16,17].

Another functional SNP is rs2476601 (+1858 C>T), involving substitution of arginine for tryptophan at codon 620 (R620 W) in the first proline-rich domain (P1) of Lyp. This variation alters the Lyp/C-Src tyrosine kinase interaction domain and results in a gain of function Lyp (increased phosphatase activity) that inhibits TCR signaling [16]. This polymorphism has been related to SLE in North America [18], RA in Mexico [19], and pSS in Colombia [20]. In the present case-control study, we investigated whether there is an association between *PTPN22* polymorphisms, their haplotypes and *PTPN22* mRNA expression and susceptibility to pSS in a Mexican population.

## 2. Materials and Methods

### 2.1. Patients and Healthy Controls

One hundred eighty healthy controls and one hundred fifty pSS patients were included in the present study. The pSS patients were classified according to the 2016 American College of Rheumatology (ACR) and European League Against Rheumatism (EURLAR) classification criteria for pSS [21]. The sample size was calculated according to the formula $n=\frac{{\left[Z\alpha \sqrt{2\hat{p}\hat{q}}+Z\beta \sqrt{p1q1+p0q0}\right]}^{2}}{{\left(p1-p0\right)}^{2}}$, and the minimum number of alleles was *n* = 283, based on the frequencies for *PTN22* +1858C>T gene polymorphism previously published in Latin-American pSS patients [20]. This study was conducted in the Hospital General de Occidente, México, and Instituto de Investigación en Ciencias Biomédicas, Universidad de Guadalajara, México. All participants were born in western Mexico with a minimum of third-generation ancestry and a Spanish-derived last name [22]. We excluded HCs with a family history of autoimmune diseases. At the time of inclusion, the pSS patients were evaluated with Sjogrën’s Syndrome Disease Activity Index (SSDAI) and Sjogrën’s Syndrome Disease Index (SSDDI) [23]. All study subjects signed informed consent. The institutional ethics and research committees approved the study under approval number: 449/16.

### 2.2. Genotyping of rs2488457 −1123 G>C, rs33996649 +788 G>A and rs2476601 +1858 C>T Polymorphisms

Peripheral blood was collected from pSS patients and HCs. Genomic DNA (gDNA) extraction was performed using Miller’s technique [24]. We used polymerase chain reaction (PCR) to identify rs2488457 (−1123 G>C), rs33996649, (+788 G>A), and rs2476601 (+1858 C>T) genotypes. The primers, enzymes, and digestive products to evaluate the SNP genotypes in our study are provided in Table 1. The forward primer for rs2488457 (−1123 G>C) contains a recognition site for the endonuclease Sac1 (GAGCTxC) with an A>G substitution (underlined) [14,25]. PCR was carried out in a final volume of 10 µL including 1× of 10× supplied buffer enzyme, 4 mM MgCl2, 2.5 mM of each dNTP, 3 mM of each primer, 0.04 units of *Taq* DNA polymerase (Invitrogen Life Technologies, Carlsbad, CA, USA) and 100 ng/μL of gDNA. The amplification protocol was as follows: initial denaturalization at 95 °C for 3 min, followed by 29 cycles of 94 °C for 30 s, 67 °C for 30 s and 72 °C for 30 s with a final extension of 72 °C for 3 min (Thermal cycler TechNet TC-5000, Cole-Palmer, Beacon Rode, ST, UK). The PCR products were digested with 3 U of *SacI* (New England Biolabs, Ipswich, MA, USA) at 37 °C for 3 h. The restriction fragments were assessed by 6% polyacrylamide electrophoresis and stained with 2% AgNO3. The products after digestion with *SacI* are shown in Table 1.

For rs33996649 (+788 G>A), PCR was carried out in a final volume of 10 µL containing 1× of supplied 10× buffer enzyme, 2.5 mM of each dNTP, 3 mM of each primer, 0.2 units of *Taq* DNA polymerase (DONGCHEN Biotech, Guangdong, China) and 100 ng/μL of gDNA. The amplification protocol was as follows: initial denaturation at 95 °C for 5 min, followed by 35 cycles of 95 °C for 40 s, 53 °C for 40 s, and 72 °C for 40 s, with a final extension of 72 °C for 5 min (Thermal cycler TechNet TC-5000, Cole-Palmer, Beacon Rode, ST, UK). The PCR product was digested with 3 U of *MspI* (New England Biolabs, Ipswich MA, USA) at 37 °C for 3 h, and the restriction fragments were observed on a 6% acrylamide gel and stained with 2% AgNO3. Table 1 show digestion products with *MspI*.

The PCR mixture for rs2476601 (+1858 C>T) was the same as for rs2488457 (−1123 G>C). The thermal cycling conditions were as follows: initial denaturation at 95 °C for 3 min, 33 cycles of denaturation at 94 °C for 30 s, annealing at 56 °C for 30 s and extension at 72°. The products were digested with 3 U of *XcmI* (New England Biolabs, Ipswich, MA, USA) at 37 °C for 3 h. The restriction fragments were separated by 6% gel polyacrylamide electrophoresis and stained with 2% AgNO3. The products after digestion with *XcmI* are shown in Table 1.

### 2.3. RNA Extraction and Reverse Transcription

Total cellular RNA was extracted from peripheral blood mononuclear cells (PMBCs) using TRIzol reagent (Invitrogen Life Technologies, Carlsbad, CA, USA) according to the manufacturer’s protocol. Repeated phenol–chloroform extraction was performed for the RNA samples, which were subjected to isolation using the Chomiczyki and Sacchi method [26]. The 260/280 ratio was used to provide an estimate of purity. Low-quality and degraded RNA samples were excluded. According to the reverse transcriptase protocol (Promega, Madison WI, USA), Oligo-Dt primers and reverse transcriptase (MMLV) were used to synthesize complementary DNA (cDNA) from 1 μg of total RNA. *PTPN22* mRNA expression was determined in twenty-eight pSS patients and twenty-eight HCs of different genotypes.

### 2.4. Quantitative PCR (qPCR)

Quantitative real-time polymerase chain reaction (qPCR) was carried out to quantify the expression of the gene of interest. The RT–qPCR protocol followed the guidelines of Minimum Information for Publication of Quantitative Real-Time PCR Experiments (MIQE) [27] using a Nano Light Cycler 2.0 (Roche Applied Science, Branford, CT, USA). Glyceraldehyde-3-phosphate dehydrogenase (*GAPDH*) was used as a reference gene to determine relative quantification after it was shown to be stably expressed in the sample [28]. The primers and hydrolysis probes were designed with Roche Universal Probe Library (*PTPN22*: cat. no. 04689011001, GAPDH: probe cat. no. 05190541001). All samples were run as duplicates. After validation of PCR efficiency for both genes, the data obtained were analyzed. A comparative threshold cycle (Cq) method with a cutoff of 40 cycles was used to determine the *PTPN22* mRNA copy number relative to GAPDH, and data are shown based on the 2^−ΔΔCq^ method [29] and 2^−ΔCq^ method [30].

### 2.5. Anti-SSA/Ro and Anti-SSB/La Serum Level Determination

Anti-SSA/Ro and anti-SSB/La serum levels were determined from serum samples stored at −80 °C until measurement using a commercially available ELISA kit (cat. no. ORG. 506 and ORG. 508, respectively, ORGENTEC Diagnostika GmbH Carl-Zeiss-Straße 49, 55129 Mainz, Germany) with a sensitivity of 1 U/mL and 0–200 U/mL standard range. A Multiskan GO spectrophotometer (Thermo Fisher Scientific Oy, Ratastie, PO, Finland) was employed to obtain the optical density of all samples. The concentration was calculated based on a standard curve, and the results are reported as U/mL. According to the ORGENTEC ELISA kit protocol, samples with values of >25 U/mL were considered positive.

### 2.6. Statistical Analysis

Concerning the evaluation of *PTPN22* gene polymorphisms, Hardy–Weinberg equilibrium (HWE) was tested using the χ^2^ test or Fisher’s exact test. Genotypic and allelic frequencies were compared by a 2 × 2 contingency table, and a χ^2^ test was performed. The Lewontin normalized coefficient D0 was used for assessing linkage disequilibrium (LD) between pairs or markers. SHEsis software was applied for haplotype analysis [31], and haplotypes with a low frequency (<1%) were not included. Student’s t test, the Mann–Whitney U test, one-way ANOVA, the Kruskal–Wallis test and Dunn’s post hoc test were applied according to the data distribution. SPSS25 (IBM Corporation; Armonk, NY, USA) and GraphPad Prism 8.0 (GraphPad Software, Incorporation; La Jolla, CA, USA) software were used for all statistical analyses. Differences were considered significant at a *p* value < 0.05 and were corrected with Bonferroni’s method according to the case. Statistical analysis to determine the fold change in *PTPN22* mRNA expression between pSS patients and HCs was performed by using the 2^−ΔΔCq^ method, and statistically significant differences were determined through the 2^−ΔCq^ method. Values were obtained using the following formulas: ^Δ^Cq = (CqPTPN22 average − CqGAPDH average) and ^ΔΔ^Cq = (^Δ^CqpSS − ^Δ^CqHC). Receiver operating characteristic (ROC) curves and the area under the ROC curve (AUC) were used to assess the performance of *PTPN22* mRNA expression level as a diagnostic tool for pSS diagnosis.

## 3. Results

### 3.1. Demographic and Clinical Characteristics

One hundred fifty pSS patients were included in this study. The average age was 55 (±10) years, and all patients were female. The disease duration was 2.3 years [interquartile range (IQR) 1–5.5], and the average lymphocytic infiltration obtained from biopsies of the minor saliva gland was 2.3 (±1.7) foci in 4 mm^2^. Anti-SSA/Ro autoantibodies were positive in 23.3% of the pSS patients and anti-SSA/La autoantibodies in 13%. SSDAI and SSDDI means were 3 (±1) and 1 (±1), respectively. The main clinical manifestations and treatments are shown in Table 2.

### 3.2. Genotype Distribution of PTPN22 rs2488457 (−1123 G>C), rs33996649 (+788 G>A), and rs2476601 (+1858 C>T) Polymorphisms

The genotypic and allelic frequencies of the rs2488457 (−1123 G>C), rs33996649 (+788 G>A) and rs2476601 (+1858 C>T) *PTPN22* polymorphisms in pSS patients and HCs and their comparison are shown in Table 3. All *PTPN22* gene polymorphisms were in Hardy-Weinberg equilibrium. Overall, genotypic and allelic frequencies for rs2488457 (−1123 G>C) in the pSS patients were similar to those in HCs (GG 52%, GC 40.7% and CC 7.3% vs. GG 52.2%, GC 40% and CC 7.8%, respectively), with no significant differences (*p* > 0.05). Similarly, for rs33996649 (+788 G>A), there were no statistically significant differences in allele and genotype frequencies between the groups (GG 96.6%, GA 2.7% and AA 0.7% vs. GG 98.3% and GA 1.7%). Regarding rs2476601 (+1858 C>T), allele and genotype frequencies were similar in pSS patients and HCs (CC 98% CT 1.3% and TT 0.7% vs. CC 98.9%, CT 1.1% and TT 0%), with no significant differences between genotypic and allelic frequencies in pSS patients compared to HCs and a very low frequency of the T allele.

### 3.3. PTPN22 rs2488457 (−1123 G>C), rs33996649 (+788 G>A), and rs2476601 (+1858 C>T) Haplotypes

rs2488457 (−1123 G>C) and rs2476601 (+1858 C>T) were found to be in medium linkage disequilibrium (LD) (D’ = 0.70). On the other hand, the loci rs33996649 (+788 G>A) did not found in linkage disequilibrium with rs2488457 (−1123 G>C) and rs2476601 (+1858 C>T). The most frequent haplotype in pSS patients and HCs was GGC (70.7% vs. 71%, respectively), which included the three wildtype alleles of the SNPs. CGC frequencies were similar in pSS (26.3%) and HC (27.73%) (*p* > 0.05) (Table 3).

### 3.4. PTPN22 mRNA Expression and Clinical Association

**Figure 1:**
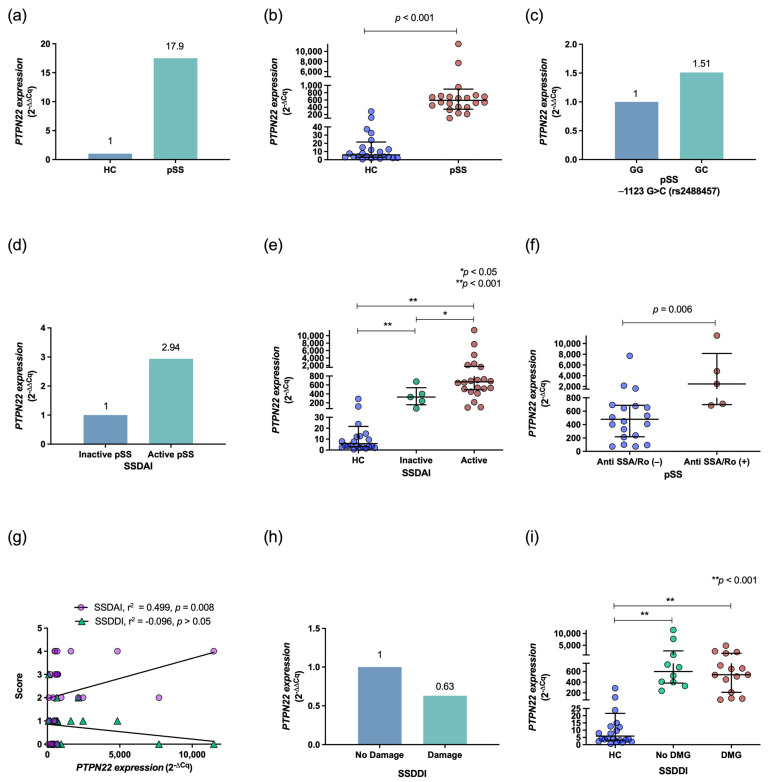
Association of *PTPN22* expression with pSS, the rs2488457 (−1123 G>C) polymorphism, and clinical and histopathological indices. Expression was higher in pSS (*n* = 28) than HCs (*n* = 28) by both qualitative and quantitative methods ((**a**) and (**b**), respectively). pSS patients with the rs2488457 (−1123 GC) (*n* = 13) genotype showed 1.51-fold more mRNA expression than rs2488457 (−1123 GG) (*n* = 15) carriers (**c**). Active pSS (*n* = 22) was associated with higher *PTPN22* expression by both qualitative and quantitative methods (**d**,**e**). pSS patients with anti-SSA/Ro+ showed high *PTPN22* mRNA expression (**f**). *PTPN22* expression showed a positive correlation with SSDAI score (**g**). mRNA expression was lower in pSS patients with damage (**h**,**i**). Qualitative gene expression data are shown through the 2^−ΔΔCq^ method. Quantitative gene expression data are shown in REU obtained through the 2^−ΔCq^ method. The *p* value was obtained through the Mann–Whitney U test or Kruskal–Wallis with Dunn’s post hoc test, as appropriate. Spearman’s rank correlation test was used. Data are shown as the median and IQR. DMG: damage, HC: healthy controls, IQR: interquartile range, pSS: primary Sjögren’s syndrome, *: *p* < 0.05; **: *p* < 0.001.

*PTPN22* expression was determined in 28 pSS patients and 28 HCs. The pSS patients showed 17.9-fold higher *PTPN22* gene expression than the HCs (Figure 1a) (*p* = 0.001, Figure 1b). When comparing *PTPN22* gene expression according to rs2488457 (−1123 G>C) genotype in the pSS group, carriers of the GC genotype showed slightly higher expression (0.51-fold more) than GG carriers; however, no significant difference was found (*p* < 0.05; see Figure 1c). In addition, patients with active pSS expressed 1.94-fold higher levels of *PTPN22* than patients with inactive pSS (Figure 1d*).* Quantitative expression of *PTPN22* was higher in pSS patients with active disease (*p* < 0.05, Figure 1e) and in those positive for anti-SSA/Ro antibodies (*p* = 0.006, Figure 1f), and a positive correlation with SSDAI was also observed (*r^2^* = 0.499, *p* = 0.008, Figure 1g). According to damage status and SSDDI score, *PTPN22* expression was similar in pSS patients (Figure 1h) but higher than that in HCs (Figure 1i, *p* < 0.001), with no statistical correlation (*r^2^* = −0.096, *p* > 0.05, Figure 1g).

**Figure 2:**
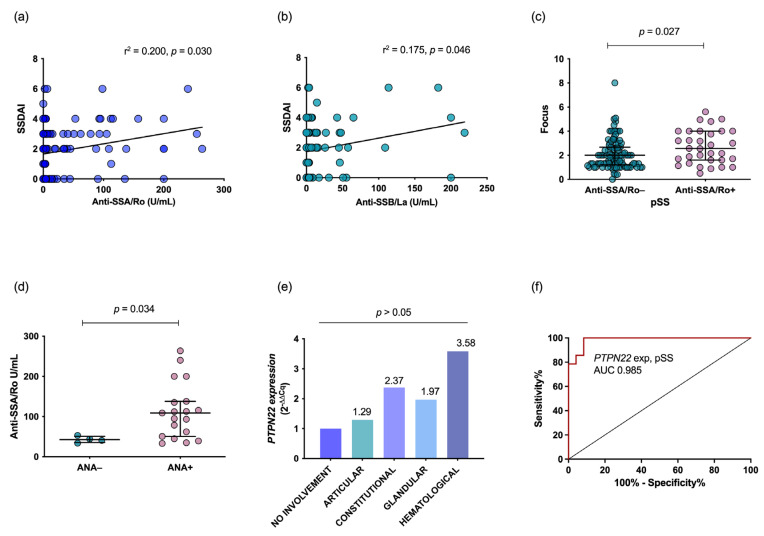
Association and correlation of autoantibodies, clinical activity and *PTPN22* expression. The SSDAI score showed a positive correlation with anti-SSA/Ro and anti-SSB/La autoantibodies (**a**,**b**). The inflammatory focus of the MSG was associated with anti-SSA/Ro+ pSS (**c**). pSS patients positive for ANA had higher anti-SSA/Ro levels (**d**). High hematological, glandular, constitutional and articular domain scores were associated with greater *PTPN22* mRNA expression (**e**). *PTPN22* gene expression was accurate for pSS diagnosis (**f**). Spearman’s rank correlation test was used. Qualitative gene expression data are shown through the 2^−ΔΔCq^ method; the *p* value was obtained by the Mann–Whitney U test using quantitative gene expression data from the 2^−ΔCq^ method. AUC was calculated through ROC analysis. Data are shown as the median and IQR. AUC: area under the curve, ANA: antinuclear antibodies, MSG: minor salivary gland, pSS: primary Sjögren´s syndrome, IQR: interquartile range, ROC: receiver operating characteristic.

Regarding clinical manifestations and autoantibody profiles, SSDAI score had a positive correlation with anti-SSA/Ro (*r^2^* = 0.200, *p* = 0.03, Figure 2a) and anti-SSB/La (*r^2^* = 0.175, *p* = 0.046, Figure 2b) serum levels. Additionally, a significantly higher focus score for MSG biopsies and ANA titers was found in anti-SSA/Ro-positive patients (*p* < 0.05, Figure 2c and Figure 2d). Patients with high SSDAI hematological domain scores showed 2.58-fold higher expression than patients with quiescent disease (Figure 2e). Furthermore, *PTPN22* expression displayed an AUC = 0.98 for accurate diagnosis of pSS (Figure 2f).

## 4. Discussion

pSS is a systemic autoimmune disorder characterized by focal lymphocytic infiltration into the exocrine glands, causing dry eyes and dry mouth [1]. It has been suggested that pSS etiology is complex; however, TCR dysregulation plays an important role in the pathogenesis of autoimmune diseases [32]. Lyp is a tyrosine phosphatase that regulates T cells through inhibitory signaling by dephosphorylating several substrates, including the Src family kinases Lck and Fyn, as well as ZAP-70, during TCR lymphocyte activation [4,33]. The Lyp protein is encoded by the *PTPN22* gene on chromosome 1. rs2488457 (−1123 G>C), rs33996649 (+788 G>A) and rs2476601 (+1858 C>T) are functional polymorphisms of the *PTPN22* gene associated with multiple inflammatory conditions, including autoimmune disorders such as pSS [7,20,33].

Our study analyzed the SNPs rs2488457 (−1123 G>C), rs33996649 (+788 G>A) and rs2476601 (+1858 C>T) in the *PTPN22* gene and susceptibility to pSS development in a Mexican mestizo population. The minor C allele of rs2488457 was detected in 27.78% of HCs, which is a lower proportion than the frequencies reported in the Asian population (33% to 41%). Nevertheless, we found a similar frequency of the rs2488457 GC genotype (40% vs. 37–46.1%) and a lower percentage of the rs2488457 CC genotype (7.8 vs. 13.7–18.1%) [10,11,12,13]. The distribution of the major rs33996649 G allele and the rs33996649 GG genotype are similar in the Mexican population [34], and the absence of the rs33996649 AA genotype is consistent with reports for European and Argentine populations [16,17,35,36]. Additionally, the minor allele frequency of rs2476601 T in the western Mexico population (0.6%) is similar to that reported in Amerindian and African populations (<1%) [7] but lower than that in Northern European populations (15%) [9]. The rs2476601 (+1858CT) genotype frequency in our study was 2.2%, lower than in European and American populations [18]. However, the rs2476601 TT genotype was absent in the Occidental Mexican population, which is consistent with previous reports for the same population [14,15,19].

Previous studies have analyzed the distribution of all these SNPs in healthy unrelated Mexican Mestizo subjects, showing genotypic and allelic frequencies similar to those reported in our study [14,15,19,35]. In general, ancestry studies in Mexican mestizos from the west region (State of Jalisco), based on maternal ancestry (mtDNA haplogroups) underscore the predominance of the Native American contribution (87%), followed by European (9%), African (3%) and Eurasian (1%) contributions [37]. However, when the Mexican admixture are analyzed based on the paternal contribution (Y-STRs), the Native American contribution decrease (28%), followed by African (5%), while the European (67%) raised [38].

rs2488457 (−1123 G>C), rs33996649 (+788 GA) and rs2476601 (+1858 C>T) were not found to be associated with an increased risk of developing pSS in the Mexican mestizo population from western Mexico. In contrast, rs2488457 (−1123 G>C) has been associated with UC, RA, and autoimmune diabetes mellitus in Asians [11,13]. The genotypic and allelic frequencies observed in west Mexican pSS patients and HCs for rs2488457 (−1123 G>C) were similar to those reported for European population and the total allelic frequencies reported in the Phase 3 of the 1000 Genomes Project [39]. Additionally, the rs2476601 T allele is associated with a risk for developing pSS in the Colombian population [20], and with RA in west [19] and central Mexican AR patients [40]. rs33996649 (+788 GA) has been reported to have a protective role against SLE and RA in European populations [16,36].

This is the first study to investigate three SNPs, rs2488457 (−1123 G>C), rs33996649 (+788 GA) and rs2476601 (+1858 C>T), in the *PTNP22* gene. The haplotype analysis showed a medium LD between rs2488457 (−1123 G>C) and rs2476601 (+1858 C>T) but not LD was found with the rs33996649 (+788 GA), and the haplotype frequencies were similar in both, pSS and HCs. Different studies evaluating *PTPN22* haplotypes with polymorphic alleles have described an increased risk of developing RA in Norway and western Mexican populations [19,41].

In addition, *PTPN22* gene polymorphisms have been associated with higher gene expression in RA and UC [13,35]. In this study, the pSS patients showed 17-fold higher mRNA expression than HCs. In another study by our group, patients with SLE showed similar *PTPN22* mRNA expression levels as controls [14]. In general, polymorphisms might explain higher gene expression. Lyp1 is mainly present in the cytoplasm of active T lymphocytes, whereas Lyp2 is found in the nucleus, perinuclear membrane, and cytoplasm of inactive peripheral T lymphocytes [42]. The third isoform reported, named PTPN22.6, lacks the catalytic site and is reported to be predominant in RA patient carriers of the rs2476601 (+1858 C>T) R620W functional variant. PTPN22.6 leads to higher nuclear factor of activated T cells (NFAT) expression and elevated IL-2 levels, with uncontrolled autoreactive T cell clonal expansion, by exerting a dominant negative effect over Lyp 1. Additionally, expression of PTPN22.6 correlates with RA activity [43]. Similar to Chang et al., we found an association between *PTPN22* mRNA expression and clinimetric indices and autoantibody profiles in RA patients, which is the most important finding of our study.

T cell receptor dysregulation is a key factor in glandular tissue damage: it is associated with a higher concentration of inflammatory cytokines [2] and promotes B cell activation, class switching, the T cell-dependent autoantibody response and germinal center (GC) expansion [44]. GC expansion has also been associated with higher production of pSS autoantibodies, such as anti-SSA/Ro, anti-SSB/La, antinuclear antibodies, and rheumatoid factor. On the other hand, murine model studies have demonstrated that *PTPN22* loss of function in myeloid cells results in an augmented inflammatory effector phase of autoimmune disease and GC generation by influencing the number and activity of Th follicular cells [44,45]. The presence of anti-SSA/Ro and anti-SSB/La correlates with severe lymphocytic infiltration of the salivary glands, a higher prevalence of extraglandular manifestations and recurrent swelling of the parotid glands [46]. In our patients with pSS, we observed a clinical association between pSS activity and damage indices, autoantibodies, and MSG infiltration.

Anti-SSA/Ro and histopathological MSG focus scores are the only two diagnostic tools used to classify pSS patients. Therefore, we evaluated *PTPN22* gene expression as a biomarker. The area under the curve of *PTNP22* expression was 0.985 (the cutoff suggested was >60 relative expression units, with 100% sensitivity, 91.67% specificity, and likelihood ratio 12; data not shown), demonstrating high diagnostic performance for pSS, which is similar to the accuracy of anti-SSA/Ro autoantibody diagnosis [47]. In populations such as ours, with a low frequency of anti-SSA/Ro (25%) antibody positivity, *PTPN22* expression may be helpful as a molecular biomarker for pSS diagnosis.

This study has important limitations as small sample size, selective recruiting of the western Mexican population, lack of inclusion of patients with the homozygous rs2488457 (−1123 CC) genotype for analysis of *PTPN22* mRNA expression, lack of inclusion of control disease for comparative analysis of *PTPN22* mRNA, as well as heterogeneity in the treatment of pSS, which may reflect differences in *PTPN22* mRNA expression. Moreover, the PTPN22.6 isoform was not evaluated.

## 5. Conclusions

In summary, the rs2488457 (−1123 G>C), rs33996649 (+788 G>A) and rs2476601 (+1858 C>T) polymorphisms of the *PTNP22* gene are not associated with the risk susceptibility of pSS in the Mexican population. We propose that *PTPN22* expression could be used as a molecular biomarker in pSS, as *PTNP22* expression is associated with autoantibody presence, disease activity index, and extraglandular manifestations. However, further studies are required to analyze interacting epigenetic factors, as well as the relationship between Lyp and the local environment of the germinal centers on exocrine glands.

## Figures and Tables

**Table 1:** Primer sequences for *PTPN22* rs2488457G>C, rs33996649G>A and rs2476601C>T polymorphisms.

SNP	Primer	Sequence	Enzyme	Products/Genotype
rs2488457 (−1123 G>C)	Forward	5′-CCA TTG AGA GGT TAT GCG AGCT-3′	*SacI*	205 pb, G/G 205, 183 and 22pb, G/C 183 and 22pb, C/C
	Reverse	5′-CAA CCA CCT TGC TGA CAA CAT TG-3′		
rs33996649 (+788 G>A)	Forward	5′-GAT GGA GCA AGA CTC AGA CAC-3′	*MspI*	234 pb A/A 234, 91 and 143 pb, G/A 91, 143pb G/G
	Reverse	5′-CCC CAT GTT AGA AGA GCA GAT-3		
rs2476601 (+1858 C>T)	Forward	5′ ATTTGCTTCAACGG AATTT-3′	*XcmI*	412 pb, C/C 412, 246 and 166bp, C/T 246 and 166 pb, T/T
	Reverse	5′-CAT GCT GCT ATT GCT CTG CT-3′		

SNP: Single nucleotide polymorphism.

**Table 2:** Demographic and clinical characteristics of the primary Sjogrën syndrome patients.

	pSS (*n* = 150)
Characteristics	
Gender, Female/Male	150/0
Age, years ^a^	55 (±10)
Disease duration, years ^b^	2.3 (1–5.5)
Inflammation markers	
ESR, mm/h ^a^	25 (±16)
CRP, mg/dL ^a^	5.04 (±4.6)
Antibodies	
Anti-Ro+, IU/mL, *n* (%)	35/127 (23.3)
Anti-La+, IU/mL, *n* (%)	20/127 (13)
Glandular tests	
Schirmer test positive, *n* (%)	120 (80)
MSG biopsy, focus ≥ 1, *n* (%)	143 (98)
Focus score ^a^	2.3 (±1.7)
Clinical domains ^c^	
Constitutional, *n* (%)	81 (54)
Glandular, *n* (%)	12 (8)
Articular, *n* (%)	62 (41.3)
Hematologic, *n* (%)	9 (6)
Vascular, *n* (%)	3 (2)
Sjögren’s syndrome indices	
SSDDI ^a^	1 (±1)
SSDAI ^a^	3 (±1)
Treatments	
Prednisone, *n* (%)	14 (10)
Azathioprine, *n* (%)	24 (17)
Methotrexate, *n* (%)	39 (27)
Antimalarials, *n* (%)	76 (57)

^a^ Data are shown as mean and standard deviation; ^b^ Data is shown as median and percentile 25–75. ^c^ Clinical domains: Constitutional, fever ≥ 38° not caused by infections, fatigue affecting normal activities and worsening of fatigue; Glandular, appearance or increased inflammation of the major salivary glands, not due to infection or stones; Articular, inflammatory pain in = 1 joints or evolving arthralgias; Hematologic, lymphopenia (<1.4 × 10^3^/µL), leukopenia (<4.0 × 10^3^/µL), or clinically palpable lymph nodes/spleen, imaging confirmed pleurisy, not caused by infection, pneumonia (segmental or interstitial) CT-confirmed cut-glass appearance, not caused by infection; Vascular, new appearance or worsening or recurrent outbreaks of palpable purpura, proteinuria > 0.5 mf/day, increased serum creatinine outside normal parameters, histologically proven glomerular or interstitial nephritis. ESR: Erythrocyte Sedimentation Rate; CRP: C-reactive protein; MSG: minor salivary glands; SSDI: Sjogrën’s Syndrome Injury Index; SSDAI: Sjögren’s Syndrome Activity Index.

**Table 3:** Genotype, allele and haplotype frequencies of *PTPN22* rs2488457G>C, rs33996649G>A and rs2476601C>T polymorphisms in pSS and HC groups.

SNP	pSS *n* = 150 (%)	HC *n* = 180 (%)	*p* Value	*Pc* Value	OR (CI 95%)
rs2488457 (−1123 G>C)					
Genotype					
GG	78 (52)	94 (52.2)	1	1	-
GC	61 (40.7)	72 (40)	0.864	1	0.927 (0.406–2.211)
CC	11 (7.3)	14 (7.8)	0.899	1	0.947 (0.426–2.189)
Allele					
G	217 (72.3)	260 (72.2)	1	-	-
C	83 (27.7)	100 (27.8)	0.974	-	0.995 (0.708–1.399)
rs33996649 (+788 G>A)					
Genotype					
GG	145 (96.6)	177 (98.3)	1	1	-
GA	4 (2.7)	3 (1.7)	0.706 *	1	1.628 (0.432–6.534)
AA	1 (0.7)	0	0.452 *	0.904	-
Allele					
G	296 (98.7)	357 (99.2)	1	-	-
A	4 (1.3)	3 (0.8)	0.708 *	-	1.608 (0.429–6.420)
rs2476601 (+1858 C>T)					
Genotype					
CC	147 (98)	178 (98.9)	1	1	-
CT	2 (1.3)	2 (1.1)	0.849 *	1	1.211 (0.188–7.801)
TT	1 (0.7)	0	0.272 *	0.544	-
Allele					
C	296 (98.7)	358 (99.4)	1	-	-
T	4 (1.3)	2 (0.6)	0.295 *	-	2.419 (0.556–12.78)
Haplotypes					
GGC	212.01 (70.7)	255.44 (71)	1	-	-
CGC	78.99 (26.3)	99.56 (27.7)	0.773	-	0.950 (0.670–1.338)

Data were analyzed with chi square test or * Fisher’s exact test when data require. Statistical difference *p* < 0.05; *P*c: *p* corrected value according to Bonferroni adjustment, *P*c = *p*-value obtained × 2 (genotypes case); SNP: single nucleotide polymorphism, pSS: primary Sjögren’s syndrome; HC: healthy controls; OR:odds ratio; CI: confidence interval.

## Data Availability

Not applicable.
